# Viability and stress protection of chronic lymphoid leukemia cells involves overactivation of mitochondrial phosphoSTAT3Ser_727_

**DOI:** 10.1038/cddis.2014.393

**Published:** 2014-10-09

**Authors:** C Capron, K Jondeau, L Casetti, V Jalbert, C Costa, E Verhoyen, J M Massé, P Coppo, M C Béné, P Bourdoncle, E Cramer-Bordé, I Dusanter-Fourt

**Affiliations:** 1 Institut Cochin, Inserm U1016, Paris, France; 2 Service d'Hématologie-Immunologie, Hôpital Ambroise Paré, Boulogne-Billancourt, France; 3 Université Paris Descartes, Sorbonne Paris Cité, Paris, France; 4 CNRS UMR8104, Paris, France; 5 Université de Versailles St Quentin en Yvelines, Guyancourt, France; 6 Ecole Normale Supérieure de Lyon, Université de Lyon, UCB-Lyon1, Lyon, France; 7 INSERM U1065, Lyon, France; 8 Service d'Hématologie Clinique, Hôpital Saint Antoine and Université UPMC, Paris, France; 9 Service d'Hématologie Biologique, Hôtel-Dieu-CHU, Nantes, France

## Abstract

Chronic lymphoid leukemia (CLL) is characterized by the accumulation of functionally defective CD5-positive B lymphocytes. The clinical course of CLL is highly variable, ranging from a long-lasting indolent disease to an unpredictable and rapidly progressing leukemia requiring treatment. It is thus important to identify novel factors that reflect disease progression or contribute to its assessment. Here, we report on a novel STAT3-mediated pathway that characterizes CLL B cells-extended viability and oxidative stress control. We observed that leukemic but not normal B cells from CLL patients exhibit constitutive activation of an atypical form of the STAT3 signaling factor, phosphorylated on serine 727 (Ser_727_) in the absence of detectable canonical tyrosine 705 (Tyr_705_)-dependent activation *in vivo*. The Ser_727_-phosphorylated STAT3 molecule (pSTAT3Ser_727_) is localized to the mitochondria and associates with complex I of the respiratory chain. This pSer_727_ modification is further controlled by glutathione-dependent antioxidant pathway(s) that mediate stromal protection of the leukemic B cells and regulate their viability. Importantly, pSTAT3Ser_727_, but neither Tyr705-phosphorylated STAT3 nor total STAT3, levels correlate with prolonged *in vivo* CLL B cells survival. Furthermore, STAT3 activity contributes to the resistance to apoptosis of CLL, but not normal B cells, *in vitro*. These data reveal that mitochondrial (Mt) pSTAT3Ser_727_ overactivity is part of the antioxidant defense pathway of CLL B cells that regulates their viability. Mt pSTAT3Ser_727_ appears to be a newly identified cell-protective signal involved in CLL cells survival. Targeting pSTAT3Ser_727_ could be a promising new therapeutic approach.

Chronic lymphoid leukemia (CLL) is characterized by the accumulation of proliferating CD5^+^ B lymphocytes.^1^ The clinical course of CLL is highly variable, ranging from a long-lasting to a rapidly progressing leukemia requiring treatment. Although the understanding of CLL pathophysiology has improved markedly in the past few years, therapeutic approaches are still not curative. It is thus important to further identify the factors that selectively sustain CLL cells survival.

CLL B lymphoid cells (CLL-BCs) have a prolonged survival time *in vivo* but exhibit a high level of spontaneous apoptosis *in vitro*, highlighting the critical role of CLL-BC microenvironment in this pathology.^1–3^ Several stromal factors have been shown to be implicated in promoting CLL-BC viability *in vitro*,^4, 5, 6^ yet the exact mechanisms by which the microenvironment protects CLL cells from apoptosis *in vivo* remain unclear.

The STAT3 signaling factor mediates numerous extracellular survival/growth messages. It is activated by phosphorylation of tyrosine 705 (Tyr_705_), allowing STAT3 to bind to DNA and activate the transcription of target genes.^7^ Abnormal constitutive activation of Tyr705-phosphorylated STAT3 (pSTAT3Tyr_705_) is observed in multiple tumor cells and contributes to oncogenic processes.^8,9^ In addition, STAT3 can be phosphorylated at serine 727 (Ser_727_) by growth factor-activated serine kinases, thereby modulating STAT3 transcriptional activity and regulating the activity of associated transcriptional factors such as NFκB.^10^ Remarkably, STAT3 was recently reported to exhibit extranuclear pro-oncogenic activities in murine cells, linked to its mono-phosphorylation on Ser_727_ but not Tyr_705_, subsequent association with mitochondrial (Mt) components and regulation of the respiratory chain.^11,12^ In 1997, Frank and Mahajan^13^ showed by western blotting a remarkable constitutive phosphorylation of STAT3Ser_727_ in the absence of canonical pSTAT3Tyr_705_ in 100% of 32 primary CLL-BC samples. Hazan-Halevy *et al.*^14^ later showed that STAT3 limited the spontaneous apoptosis of CLL-BC *in vitro*.

In the present study, we addressed the role of Ser727-phosphorylated STAT3 (pSTAT3Ser_727_) in CLL pathophysiology. We demonstrate that circulating CLL-BCs but not normal B lymphoid cells (N-BCs) overexpress an atypical mitochondria-associated pSTAT3Ser_727_ moiety, the level of which correlates with prolonged CLL-BC survival *in vivo*. We further identified glutathione (GSH), an antioxidant mediating stromal protection of the leukemic cells, to be a critical regulator of Ser_727_ phosphorylation, and provide evidence that STAT3 is responsible for the extended survival of CLL-BC as compared with N-BC. Overall, our data suggest that Mt pSTAT3Ser_727_ contributes to the protection of CLL-BC. As such, it may represent a new biochemical pathway for effectively targeting leukemia cells, especially those that exhibit drug resistance.

## Results

### Overactivation of pSTAT3Ser_727_ correlates with CLL-BC resistance to apoptosis

**Figure 1:**
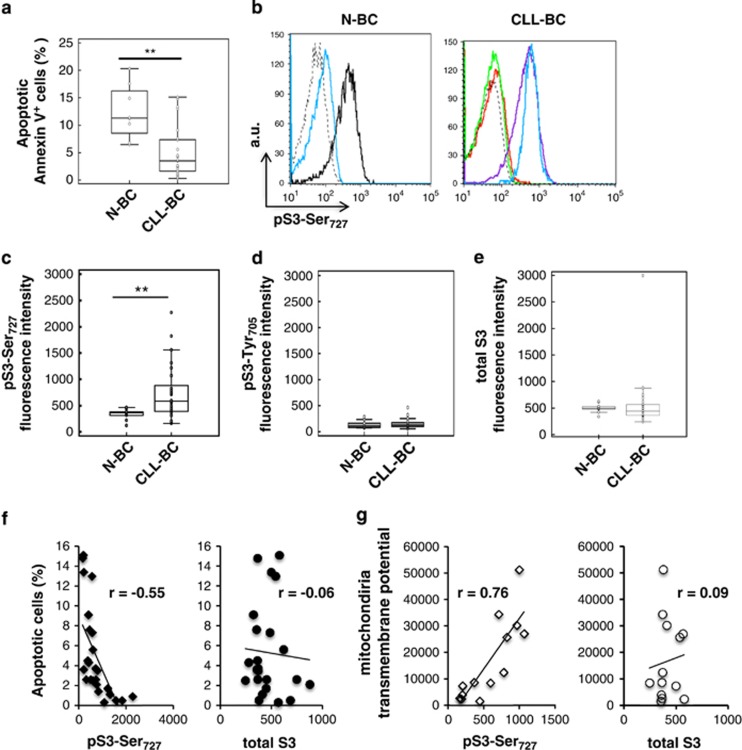
Activation of pSTAT3Ser727 correlates with CLL-BC resistance to apoptosis. (**a**) Flow cytometry measurement (FCM) of B-cell apoptosis by Annexin V staining of CD45^+^CD19^+^ normal (N-BC) and CLL (CLL-BC) B cells. (**b**–**e**) FCM of pSTAT3Ser_727_ (**b** and **c**), STAT3Tyr_705_ (**d**) and total STAT3 (**e**) of N-BC and CLL-BC. (**b**) One representative experiment is shown. B cells were labeled with rabbit control (dashed black line), total STAT3 or pSTAT3Ser_727_ antibodies, as indicated, in the absence (blue line) or presence of pSTAT3Ser_72_ immunogen peptide (ipep; green line), phosphorylated S3Ser_727_ 11-peptide (red line) or unphosphorylated S3Ser_727_ 11-peptide (purple line). The black line indicates pSTAT3Ser_727_ labeling following a 15-min treatment of N-BC with phorbol myristyl acetate. Results are expressed as mean fluorescence intensity (arbitrary unit, a.u.). (**c**–**e**). Statistical analyses of the indicated immunolabeling mean fluorescence intensities are shown (N-BC, *n*=8; CLL-BC, *n*=29; **P<0.01). (**f** and **g**) Correlation between pSTAT3Ser_727_ or total STAT3 levels (mean fluorescence intensity) and the percentage of apoptotic Annexin V-positive cells (**f**) or DiOC6-probed mitochondrial transmembrane potential (G), evaluated in CLL-BC samples (*r*, Pearson's coefficient; *n*=29)

Circulating B cells were purified from healthy donors (*n*=8) and CLL patients (Supplementary Table 1). Samples were studied immediately upon collection to avoid any culture artifacts owing to the spontaneous apoptosis of these cells *in vitro*. Apoptosis was measured by combining Annexin V/7-AAD (7-aminoactinomycin D) staining of phosphatidyl serine externalization and dead cell DNA labeling. Apoptosis was also assessed by monitoring the Mt transmembrane potential (MTP) that characterizes mitochondria in viable cells. CLL-BC showed significantly lower apoptosis than N-BC using both assays, consistent with the extended survival of CLL-BC *in vivo* (Figure 1a and data not shown). The intensity of this labeling was variable from patient to patient, which is in agreement with the heterogeneity of CLL disease. STAT3 phosphorylation was investigated by flow cytometry measurements (FCMs). As shown in Figure 1b, CLL-BC showed strong immunolabeling using an anti-pSTAT3Ser_727_ antibody. This labeling was abolished by the pSTAT3Ser_727_ immunogen peptide (ipep) as well as by an 11 amino-acid-long STAT3Ser_727_ phosphopeptide but not by the unphosphorylated peptide, thus confirming labeling specificity. Conversely, quite low pSTAT3Ser_727_ immunolabeling was detected in N-BC under similar conditions, although pSTAT3 Ser_727_ was normally induced by phorbol esters in these cells (Figure 1b, left panel). Statistical analyses confirmed that circulating CLL-BC expressed higher levels of pSTAT3Ser_727_ as compared with N-BC (Figure 1c).

Regarding pSTAT3Tyr_705_, CLL-BC and N-BC showed a similar weak-to-undetectable immunolabeling (Figure 1d). In addition, CLL-BC and N-BC expressed the same total STAT3 levels (Figure 1e). Western blot analysis of the same cells confirmed that CLL-BC overexpressed pSTAT3Ser_727_ as compared with N-BC in the absence of detectable pSTAT3Tyr_705_. Also, no activation of the related STAT5 factor was observed (Supplementary Figure 1).

The highly variable course of CLL led us to compare pSTAT3Ser_727_ levels with the apoptosis indexes of CLL-BC. A significant negative correlation was observed between pSTAT3Ser_727_ level and the percentage of apoptotic Annexin V-positive CLL-BC (Figure 1f, right, *n*=29). Conversely, pSTAT3Ser_727_ levels showed a positive correlation with the mitochondria transmembrane potential (MTP) that labeled viable cells (Figure 1g, right, *n*=29). Notably, no correlation was observed between total STAT3 levels and CLL-BC basal apoptosis using both criteria. These data indicate that CLL-BC-extended survival correlates with pSTAT3Ser_727_ activation in the absence of pSTAT3Tyr_705_ activation and total STAT3 amplification. This suggests that pSTAT3Ser_727_ contributes to CLL-BC leukemic properties *in vivo*.

### pSTAT3Ser_727_ localizes to CLL-BC mitochondria

**Figure 2:**
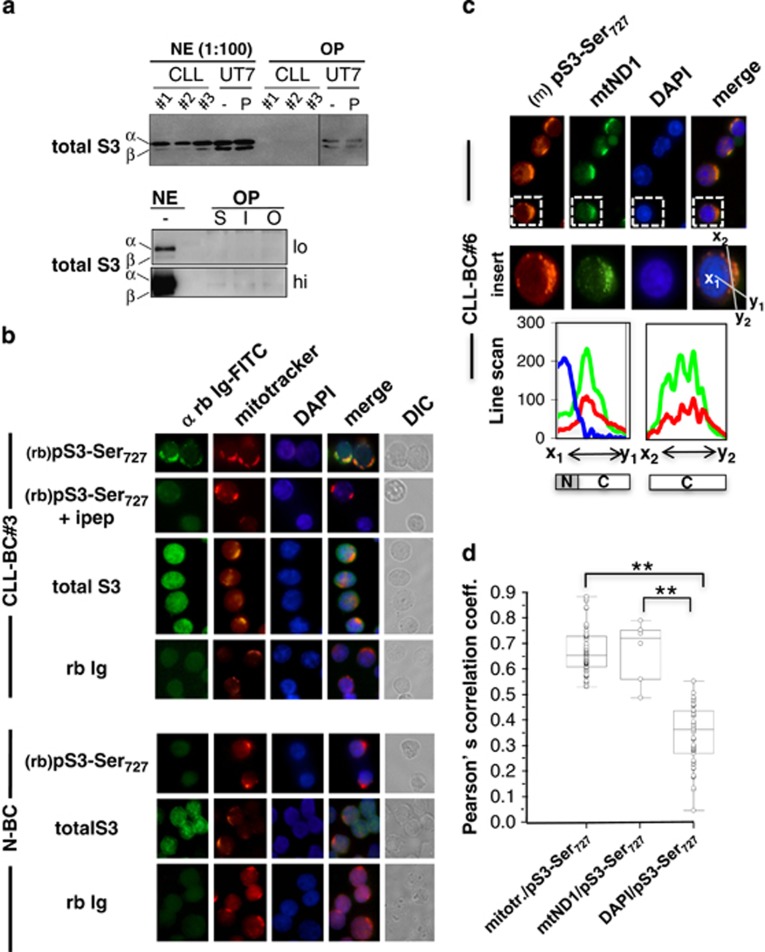
pSTAT3Ser_727_ subcellular localization characterizes human primary CLL B cells. (**a**) Nuclear extracts from CLL-BC (#*n* upper panel, lower panel) or UT7 hematopoietic cell line were prepared and incubated with biotin-labeled STAT3-binding site DNA probes and streptavidin magnetic beads for pull-down assays. Attached proteins were separated by SDS-PAGE and STAT3 was detected by immunoblotting. NE and OP indicate nuclear extracts and oligo-pull-down extracts, respectively; 1:100 of total NE were loaded on each gel. NE were incubated with biotin-labeled SIEm67- (upper panel) or with SIEm67 (S), IRF (I)- or Oct (O)-DNA (lower panel) probes; lo and hi indicate low and high exposure of the immunoblot. UT7 hematopoietic cells were pre-incubated for 15 min in the absence or presence of phorbol myristyl acetate (P) as positive controls. *α* and *β* indicate STAT3*α* and STAT3*β* isoforms. (**b**–**d**) CLL-BC and N-BC were isolated, fixed, permeabilized and processed for fluorescence confocal microscopy as indicated. (**b**) Rabbit (rb) anti-pSTAT3Ser_727_ (pS3Ser_727_) antibody in the absence or presence of immunogen peptide (ipep), rabbit anti-total STAT3 (total S3) and isotype control rabbit immunoglobulins (rb Ig) were used in co-labelings with mitotracker, as indicated. DIC indicates differential interference contrast. (**c**) Mouse anti-pSTAT3Ser_727_ (pS3Ser_727_ mAb) and rabbit anti-mtND1 antibodies were used in co-labeling assays. Fluorescent probes were FITC-coupled anti-rabbit Ig (rb Ig-FITC, green), red-mitotracker or PE-coupled anti-mouse Ig (red), DAPI (blue). Line scan intensity profiles (bottom) of pS3Ser (red), mtND1 (green) and DAPI (blue) labeling along indicated axes (*x*_1_–*y*_1_; *x*_2_–*y*_2_) from gated areas. DAPI-positive nuclear (N) and DAPI-negative cytoplasmic (C) areas are schematized. Representative images from five patients (#*n* relates to Table I patient's number) and five healthy donor samples are shown. Magnification, × 100. (**d**) Pearson'correlation coefficients were measured for the indicated fluorescent signals (rabbit anti-pSTAT3Ser_727_ *versus* mitotracker; mouse anti-pSTAT3Ser_727_ *versus* rabbit anti-mtND1; mouse anti-pSTAT3Ser_727_ *versus* DAPI). Twenty-five cells were randomly examined in confocal microscopy for each patient, and data from five and three patients were pooled, which corresponds to over 100 cells observed; **P<0.01)

A single serine phosphorylation was shown to mediate STAT transcription activation.^15^ We determined whether CLL-BC pSTAT3Ser_727_ binds DNA. CLL-BC nuclear extracts were prepared and pull-down experiments using biotin-labeled STAT3-DNA probes were performed. pSTAT3Ser_727_ was mostly undetectable in the nuclear fraction. Neither STAT3 nor pSTATSer7227 did bind STAT3-DNA elements (Figure 2a and data not shown). pSTAT3Ser_727_ localization was next examined using immunolabeling coupled to confocal microscopy. In CLL-BC, pSTAT3Ser_727_ distributed to the cytoplasm with a clear granular pattern but no significant nuclear labeling (Figure 2b, top). All fluorescence was suppressed when the rabbit anti-pSTAT3Ser_727_ antibody was pre-mixed with an excess amount of ipep (Figure 2b) or with pS3-11pep but not with the unphosphorylated STAT3 (S3)-11pep (Supplementary Figure 2). Compared with CLL-BC, N-BC showed weak-to-undetectable pSTAT3Ser_727_ labeling (Figure 2b). Immunolabeling of total STAT3 showed a rather uniform cellular distribution in CLL-BC and N-BC (Figure 2b). The peculiar intracellular distribution of pSTAT3-Ser_727_ suggests an association with cytoplasmic organelles. Because of the reported Mt activities of STAT3,^12^ the important involvement of mitochondria in cell survival and the increased MTP of CLL-BC (Figure 1), we focused on these organelles. Mitotracker, a selective dye that concentrates into mitochondria along MTP, was first used to visualize these organelles. pSTAT3-Ser_727_ was observed within Mitotracker-positive subcellular areas in co-labeling assays (Figure 2b). An additional experiment was performed using a murine monoclonal anti-pSTAT3Ser_727_ antibody that confirmed the predominant granular cytoplasmic localization of pSTAT3Ser_727_ in CLL-BC (Figure 2c). Mitochondria were co-labeled by antibodies directed to the Mt electron transfer chain (ETC) complex I NADH-ubiquinone reductase subunit (mtND1).^16^ pSTAT3-Ser_727_ immunolabeling again co-localized with this second Mt marker, as assessed by line-scan fluorescence quantification (Figure 2c). Co-labeling was further ascertained at the pixel level using Pearson's correlation coefficient measurement that validated pSTAT3Ser_727_/mitochondria co-localization statistically in patient samples (Figure 2d).

**Figure 3:**
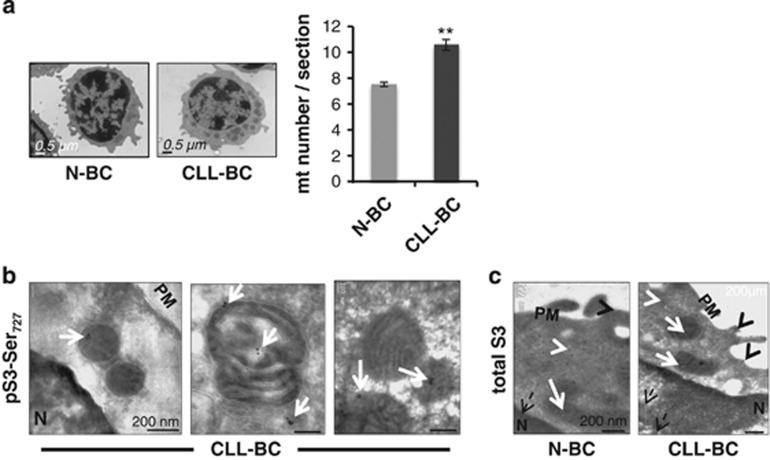
pSTATSer_727_ associates with mitochondria of CLL-BC but not N-BC. (**a**) CLL-BC (*n*=10) and N-BC (*n*=2) were processed for transmission electron microscopy (TEM) as described. Left panel: representative images of B-cell ultrastructures. Right panel: quantification of mitochondria numbers per TEM cell section. Data are presented as mean±S.E.M., (**P<0.01). (**b** and **c**) CLL-BC (*n*=3) and N-BC (C, *n*=2) were fixed, permeabilized and processed for immunogold labeling and TEM using rabbit pSTAT3Ser_727_ (**b**) or total STAT3 (**c**) antibody, as indicated. White and black arrow heads, respectively, indicate cytosol and plasma membrane (PM)- localized signals; dashed black arrows indicate nuclear (N) signals; white arrows indicate signals associated to mitochondria

B lymphocytes are small cells with a very limited cytoplasmic area. pSTAT3Ser_727_ intracellular distribution was further analyzed using transmission electron microscopy. As shown in Figure 3a, CLL-BC contained significantly more mitochondria as compared with N-BC (10.6±0.45 *versus* 7.5±0.17, *P*<0.01), which filled most of the cytoplasmic area. Immunogold labeling, performed on ultrathin sections, revealed the presence of immunoreactive pSTAT3Ser_727_, predominantly localized in CLL-BC mitochondria (Figure 3b). No signal was detected in N-BC nor in CLL-BC stained in the presence of the ipep (data not shown). By contrast, immunolabeling of total STAT3 revealed the presence of STAT3 moieties in all cell compartments, including mitochondria, in both CLL-BC and N-BC (Figure 3c).

**Figure 4:**
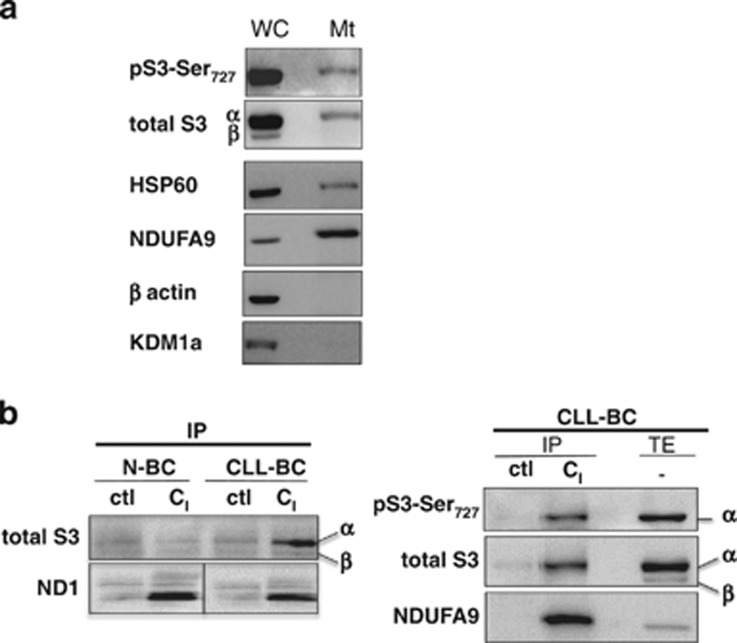
pSTATSer_727_ associates with complex I of the mitochondria respiratory chain. (**a**) Mitochondria (Mt) were isolated from CLL-BC (whole cells, WC) by magnetic immunolabeling. Extracts were separated by SDS-PAGE and blots were probed with the indicated antibodies. (**b**) Antibody to complex I (C_I_) of the ETC or a nonspecific isotype-matched IgG (ctl) were incubated with CLL-BC or N-BC total cell extracts (TEs), as indicated. Immunoprecipitates (IPs) were resolved on SDS-PAGE and probed for the indicated proteins. 1 : 50 of TE was loaded on the gel. *α* and *β* indicate STAT3*α* and STAT*β* isoforms, respectively

pSTAT3Ser_727_ intracellular localization was also assessed by subcellular fractionation. Mitochondria were isolated from cell lysates by magnetic labeling with antibodies directed to the translocase of the outer Mt membrane 22 (TOM22). Mitochondria enrichment was evaluated by SDS-polyacrylamide gel electrophoresis (SDS-PAGE) coupled to immunoblotting using the Mt heat-shock protein 60 (hsp60) and NADH dehydrogenase 1 alpha subunit 9 (NDUFA9), a component of complex I of the ETC, cytoplasmic *β*-actin and nuclear lysine demethylase 1a (KDM1a; Figure 4a). STAT3 and pSTAT3Ser_727_ associate with *β*-actin- and KDM1a-negative mitochondria-enriched fraction. Comparison of the amounts of STAT3 and hsp60 in TOM22-positive and -negative fractions indicated that levels of Mt STAT3 were ~1–5% that of total STAT3. First, evidence for the Mt localization of STAT3 has come from observations that GRIM-19, a component of the ETC complex I, directly binds to STAT3.^12^ Using an antibody that captures all components of complex I and immunoprecipation assays, we co-precipitated serine phosphorylated STAT3 in CLL-BC, but not in N-BC extracts (Figure 4b).

Collectively, these data concur in indicating that CLL-BC display overactivation of an atypical Mt pSTAT3Ser_727_ moiety.

### CLL-BC oxidative stress regulates Mt pSTAT3Ser_727_ activation

**Figure 5:**
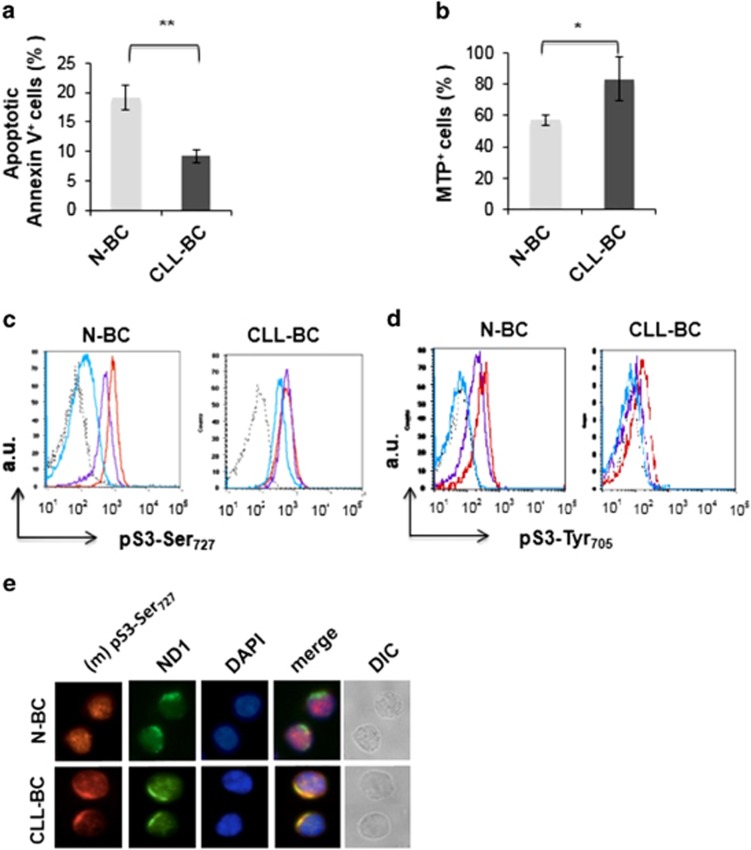
Stromal protection of CLL-BC sustains Mt pSTAT3Ser_727_ overactivation. (**a** and **b**) FCM of apoptosis (**a**) and mitochondrial transmembrane potential (MTP; **b**) of N-BC and CLL-BC at day 8 of MS5 co-culture. Results are expressed as percentage of AnnexinV-positive (**a**) and TMRM-positive (MTP^+^; **b**) cells. The mean +/− S.E.M. of 3 independent experiments is shown, *P<0.05; **P<0.01. (**c** and **d**) FCM analysis of pSTAT3Ser_727_ (**c**) or pSTAT3Tyr_705_ (**d**) expression in N-BC and CLL-BC upon MS5 co-culture. CD45^+^CD19^+^ CD5^+^ B cells were analyzed at day 0 (blue), day 3 (purple) or day 8 (red) of MS5 co-culture. Standard rabbit immunoglobulins were used as control at day 8 (dashed black line). One representative experiment is shown (samples from three healthy donors and three individual patients were analyzed). Results are expressed as mean fluorescence intensity (arbitrary unit, a.u.). (**e**) N-BC and CLL-BC were collected at day 8 of MS5 co-culture. Immunofluorescence analyses were performed with the indicated primary antibodies using fluorescence microscopy (magnification × 100). Fluorescent probes were FITC-coupled anti-rabbit Ig (green), PE-coupled anti-mouse Ig (red) and DAPI (blue). DIC indicates differential interference contrast. Representative images from three CLL patients and five healthy donors are shown

*In vivo*, their microenvironment has a profound effect on CLL-BC survival. Stromal support of CLL-BC can be reproduced when they are co-cultured with bone marrow stromal cell lines.^6,17^ Indeed, murine MS5 and human HS5 cells significantly reduce the level of CLL-BC spontaneous apoptosis, as assessed by Annexin V labeling and MTP probing (Figures 5a and b and Supplementary Figure 3). It similarly maintains a strong level of pSTAT3Ser_727_ immunolabeling in absence of any immunoreactive pSTAT3Tyr_705_ in CLL-BC even after prolonged periods (Figures 5c and d, right panels, and Supplementary Figure 3), while the expression of total STAT3 remains unchanged (data not shown). Under similar conditions, stromal cells enhance not only pSTAT3Ser_727_ but also pSTAT3Tyr_705_ activation of N-BC in a time-dependent manner.

pSTAT3Ser_727_ localization was assessed in this model. Using again two different pSTAT3Ser_727_ antibodies and mitochondria markers, CLL-BC were shown to display the same predominant Mt localization of pSTAT3Ser_727_ upon MS5 co-culture, as compared with freshly isolated CLL-BC (Figure 5e and data not shown). Conversely, a pronounced difference was observed in stroma co-cultured N-BC in which pSTAT3Ser_727_ was distributed to the cytoplasmic and nuclear compartments, and thus not predominantly associated to mitochondria.

Collectively, these observations indicate that stromal cells mimic *in vitro* the interactions between CLL-BC and their microenvironment that occur *in vivo*, in terms of CLL-BC protection from apoptosis, STAT3 phosphorylation status and intracellular localization. They further reveal that the same stromal messages are differentially received and translated by N-BC and CLL-BC, thereby leading to mitochondria-restricted pSTAT3Ser_727_ overexpression in leukemic cells only.

**Figure 6:**
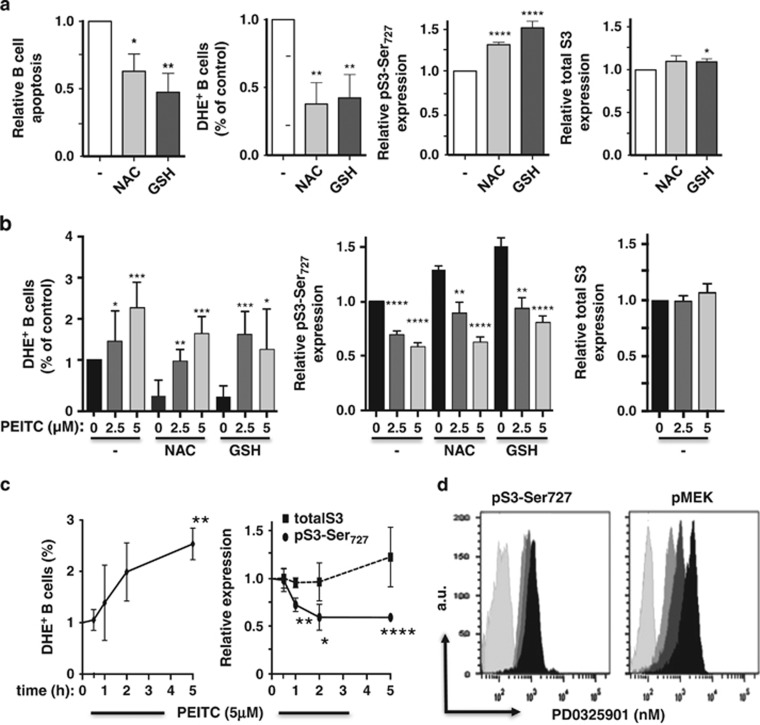
GSH metabolism regulates the phosphorylation of STAT-Ser_727_ of CLL-BC. (**a** and **b**) Comparison of apoptosis, ROS, pSTAT3Ser_727_ and total STAT3 expression of CLL-BC cultured alone for 4 days in the presence or absence of NAC (1 mM) or GSH (2 mM). Where indicated, PEITC (1–5 *μ*M, 5 h) was added at the end of the culture. (**c**) Time course of ROS, pSTAT3Ser_727_ and total STAT3 expression of CLL-BC cultured alone in the presence of PEITC (5 *μ*M) for the indicated time. ROS were measured by DHE staining of CD45/CD19^+^/CD5^+^ cells; pSTAT3Ser_727_ and total STAT3 levels were evaluated upon immunolabeling/FCM. Data are expressed as relative to untreated cells. The mean±S.E.M. of four separate experiments with four different CLL patient samples are shown (*P<0.05; **P<0.01; ***P<0.001; ****P<0.0001). (**d**) FCM of pMEK (right) and pSTAT3Ser_727_ (left) of CLL-BC upon a two-day culture in the absence (black) or presence of 10 nM (dark grey) or 100 nM (medium grey) of MEK inhibitor PD0325901. B cells were labeled with rabbit control (light grey), pMEK or pSTAT3Ser727 antibodies, as indicated. Results are expressed as mean fluorescence intensity (arbitrary unit, a.u.)

The accumulation of Mt pSTAT3Ser_727_ in stroma-supported CLL-BC led us to search for stromal signals that would regulate pSTAT3Ser_727_ activation in these cells. CLL-BC intrinsically have high levels of reactive oxygen species (ROS) when compared with normal lymphocytes.^1,18^ This makes CLL-BC more dependent on such cellular antioxidants as GSH. A critical metabolic interaction between CLL-BC and bone marrow stromal cells was recently reported to enhance GSH synthesis, and thus increase the ability of CLL-BC to maintain the redox balance and promote cell survival.^6^ We tested the relationship between GSH, oxidative stress and pSTAT3Ser_727_ expression of CLL-BC. Dihydroethidium (DHE) provides a clear indication of primary ROS levels^19^ by binding to superoxide anions that are produced predominantly by the respiratory chain of mitochondria in most cells except phagocytes. As shown in Figure 6a, the addition of GSH to CLL-BC culture medium, in absence of stromal cells, indeed enhanced ROS detoxification, thereby lowering superoxide anions levels and promoting cell survival. It further enhanced pSTAT3Ser_727_ expression of CLL-BC without changing pSTAT3Tyr_705_ activation (data not shown) and total STAT3 levels. Similar data were obtained upon addition of N-acetylcysteine (NAC), another antioxidant. This increased activation of pSTAT3Ser_727_ by antioxidants targeted Mt STAT3, as assessed by immunolabeling coupled to confocal microscopy (data not shown).

Conversely, the viability of CLL cells can be decreased by depletion of GSH using *β*-phenylethyl isothiocyanate (PEITC), a natural compound capable of inactivating cellular GSH and modulating ETC activity. PEITC indeed significantly enhanced intracellular ROS levels of CLL-BC (Figure 6b). It also decreased CLL-BC levels of pSTAT3Ser_727_ in a concentration-dependent manner while it had no impact on total STAT3 expression. It further suppressed GSH- and NAC-dependent regulation of ROS and pSTAT3Ser—but again not total STAT3–expression. Notably, the inhibitory activity of PEITC was time dependent, with a significant pSTAT3Ser_727_ loss being detectable at 1 h of treatment. This effect was observed when CLL-BCs were cultured alone (Figure 6c) or with stromal cells (data not shown), and were detectable before substantial ROS increase. These data suggest that the oxidative stress status of CLL-BC regulates pSTAT3Ser_727_. ROS directly affects the activity of a large number of cellular proteins. It also regulates the amounts of reduced GSH, which in turn positively or negatively modulate protein activity through direct conjugation, that is, glutathionylation, as observed for such Ser/Thr kinases as MEK and JNK, which may mediate GSH-dependent Mt STAT3Ser_727_ phosphorylation.^20^ The MEK–ERK pathway is required for Ras-induced phosphorylation of Mt STAT3 on Ser_727_, which is one of the critical steps of the Ras-MEK–ERK axis during transformation.^21^ Pharmacological inhibition of MEK, however, did not affect serine phosphorylation of STAT3 in CLL-BC (Figure 6d).

Altogether, these data suggest that the oxidative stress status of CLL-BC regulates pSTAT3Ser_727_ activation through a MEK–ERK independent pathway.

### STAT3 sustains CLL-BC survival

**Figure 7:**
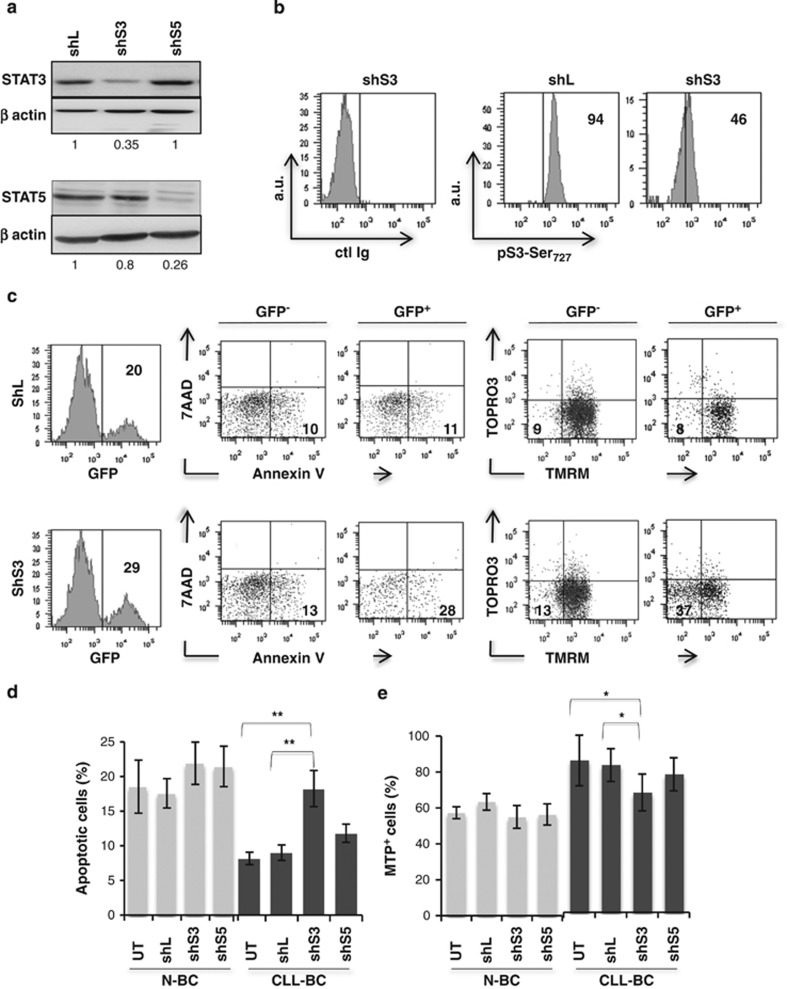
CLL-BC depends on STAT3 activity for their growth. (**a**) Immunoblot analysis of the indicated proteins in shRNA/GFP^+^ Raji B cells at day 8 post transduction. shL, shS3 and shS5 refer to control luciferase, STAT3 and STAT5 shRNA, respectively. Numbers at the bottom of each panel indicate STAT3/ *β*-actin and STAT5/*β*-actin ratio relative to shL cells, respectively. (**b**) FCM of pSTAT3Ser727 expression in CLL-BC at day 8 post transduction with the indicated shRNA/GFP encoding lentiviral vectors. B cells were co-cultured with MS5 stromal cells since day 1 post transduction. Vertical bars delineate specific fluorescent signals compared with control isotype immunoglobulins (ctl Ig, left panel). Results are expressed as mean fluorescence intensity (arbitrary unit, a.u.). Percentages of pSTAT3Ser_727_/GFP^+^ cells are indicated inside each graph. (**c**–**e**) N-BC and CLL B cells were transduced with the indicated shRNA/GFP encoding lentiviral vectors. Apoptosis was analyzed at day 8 post transduction, on GFP^+^ and GFP^-^ B cells by FCM using Annexin V/7-AAD and TMRM/TOPRO3 labeling, as indicated. (**c**) Representative experiment (*n*=10), numbers indicate the percentages of cells in relevant quadrants. (**d** and **e**) Statistical analyses expressed as percentages of AnnexinV positive (**d**) and TMRM-probed MTP positive (MTP^+^) (**e**) cells. UT indicates untransduced cells (mean±S.E.M., CLL-BC, *n*=10, N-BC, *n*=5; **P<0.01; *P<0.05)

The role of STAT3 in mediating the protection of CLL-BC was next investigated by an RNA interference-based strategy. Small hairpin RNA (shRNA) that knocks down human STAT3 (shS3) was stably introduced in N-BC and CLL-BC, using a lentiviral vector that co-encodes the green fluorescent protein (GFP) dye. Lentiviral particles that expressed measles virus glycoprotein envelope instead of canonical vesicular stomatitis virus glycoprotein were used to allow efficient transduction of human B cells, as we reported previously.^22^ shRNA targeting luciferase (shL) or STAT5 (shS5) were used as controls. As shown in Figures 7a and b, shS3 decreased *STAT3* gene expression, with no impact on STAT5 levels as compared with control shRNA (shL)-expressing human RAJI B cells, which validated the shS3 specificity. This was confirmed on CLL-BC by FCM. Transduced CLL-BC were maintained on MS5 stromal cells and the level of apoptosis of GFP^+^ and GFP^−^ subsets were analyzed along the period of culture, using AnnexinV labeling and tetramethylrhodamine-methyl-ester (TMRM) MTP probing, together with dead cell DNA staining by 7AAD or TOPRO-3 dyes. By all criteria, shS3^+^/GFP^+^ CLL-BC exhibited enhanced apoptosis as compared with shS3^-^/GFP^-^ and shL^+^/GFP^+^ control cells, whereas the viability of shS5^+^/GFP^+^ CLL-BC was unchanged (Figures 7c–e). Notably, shS3 suppressed the enhanced viability of CLL-BC, whereas it had no consequences on N-BC under similar conditions (Figures 7d and e). These data indicate that STAT3, but not STAT5, selectively supports CLL-BC survival in the absence of canonical pSTAT3Tyr_705_ activation.

## Discussion

In the present study, we investigated the physiopathological role of STAT3 phosphorylation in the natural history of CLL disease. Using human primary B cells from healthy donors and CLL-BCs from patients, this study shows that CLL-BC but not N-BC exhibit an over-activation of an atypical mitochondria-associated pSTAT3Ser_727_ moiety that characterizes leukemic cells survival and oxidative stress control. Four key results support these conclusions. First, circulating CLL-BC spontaneously express an abnormally high level of the mitochondria-restricted pSTAT3Ser_727_ moiety in the absence of canonical pSTAT3Tyr_705_ activation, at variance with what is observed in N-BC *in vivo*. Second, stroma-supported CLL-BCs, but not N-BCs, exhibit overactivation of the Mt pSTAT3Ser_727_ moiety *in vitro*. Third, both inhibitors and activators of the antioxidant GSH pathway, which regulates the redox balance and survival of CLL-BC, similarly regulate CLL-BC Mt pSTAT3Ser_727_. Fourth, the extended survival of CLL-BC correlates with their pSTAT3Ser_727_ status but not overall STAT3 expression level *in vivo* and rely on STAT3 activity, as opposed to what was observed in N-BC.

Several reports have shown that pSTAT3Ser_727_ activation in the absence of canonical pSTAT3Tyr_705_ mediates cell survival messages, as illustrated by the action of neutrotrophins on both neuronal stem/progenitor and mature cells, or VEGF activity on CLL-BC cultured alone.^23, 24, 25, 26^ DNA damage inducers similarly activate pSTAT3Ser_727_ but not pSTAT3Tyr_705_ expression in cancer cell models, thereby enhancing DNA repair.^27^ In these analyses, pSTAT3Ser_727_ activity involved nuclear transcriptional regulation of cell survival/DNA repair genes. Our study reveals that CLL-BC exhibit a unique Mt pSTAT3Ser_727_ overactivation not observed in N-BC. Wegrzyn *et al.*^12^ have indicated that Mt pSTAT3Ser_727_ enhanced mitochondria respiration of murine normal primary B cells, yet without impacting their survival. It however protected murine cardiomyocytes from ischemia and decreased the production of ROS and cytochrome-*c* release.^28^ Also, Mt pSTAT3Ser_727_ is involved in nerve growth factor-induced neurite outgrowth and the production of ROS.^29^ Remarkably, Mt pSTAT3Ser_727_ is overexpressed by Ras-transformed mouse embryo fibroblasts and supports Ras-dependent malignant transformation.^11^ It further influences the tumorigenic potential of murine 4T1 breast cancer cells that correlates with the regulation of ROS concentrations.^30^

Here our observations consistently disclosed that human primary CLL-BC spontaneously exhibit overactivation of Mt pSTAT3Ser_727_ as compared with their normal counterparts, a feature that is linked to their extended survival and oxidative stress control. To our knowledge CLL is the first human malignancy linked to a non-canonical mitochondria-restricted pSTAT3Ser_727_ overactivity *in vivo.* One prominent biochemical feature of CLL-BC is their high level of ROS and oxidative stress, when compared with normal lymphocytes. This ROS stress renders them dependent on proper redox balance and makes them oversensitive to agents that induce further ROS stress.^31,32^ Interestingly, we observed that CLL-BC exhibit an abnormal accumulation of Mt mass, which is in agreement with the data reported by Carew *et al.*^18^ The deregulated mitochondria biogenesis in CLL-BC is significantly related to endogenous ROS levels and further correlates with enhanced drug resistance.^18^ GSH is the most abundant antioxidant in cells and significantly affect cell survival.^33^ Indeed, CLL-BC from patients exhibit deficient GSH synthesis and rely on stromal cells to provide cysteine for GSH production.^6^ Our study reveals that the oxidative stress status of CLL-BC, which is controlled by cell microenvironment and dictates leukemic cell apoptosis resistance, also regulates the activation of Mt pSTAT3Ser_727_ in CLL-BC in a dose-dependent and rapid manner (<1 h), and also in absence of the active MEK–ERK pathway. It indicates that various signaling pathways can modulate serine phosphorylation of Mt STAT3 in tumor cells.

We have tested a number of inhibitory agents to decipher which kinase(s) might phosphorylate STAT3Ser_727_ of CLL-BC. They included PI3k inhibitors (Ly294002, Wortmanin), mTOR inhibitors (rapamycin and AZD8055) and many others (data not shown). GSH activators and inhibitors were the very first ones to affect pSTAT3Ser_727_ of CLL-BC. Clearly, more work needs to be done for pSTAT3Ser_727_ mode of activation in CLL-BC. Our observation that the GSH antioxidant pathway regulates pSTAT3Ser_727_ expression questions whether pSTAT3Ser_727_ effectors may influence ROS production. pSTAT3Ser_727_ associates with complex I of CLL cells. In the presence of increased ROS, STAT3 can be oxidized to form multimers.^34^ An alternative oxidative modification of STAT3 by S-glutathionylation has also been observed.^35^ Complex I-associated STAT3 of CLL-BC may thus limit ROS production by decreasing electron leak of complex I and/or by serving as a critical electron scavenger, as previously suggested.^28, 29, 30^

*In vivo*, the microenvironment has a profound effect on CLL-BC survival. Besides BCR signaling, several stromal factors have been implicated in sustaining CLL-BC survival. A number of these factors activate not only pSTAT3Tyr_705_ but also pSTAT3Ser_727_ in N-BC and CLL-BC when added to culture medium separately in the absence of stromal cells.^4,5,23^ Our study now reveals that the mixed protective signals provided by stromal microenvironments *in vivo* and *ex vivo* (i.e., MS5/HS5) are all combined to a unique mitochondria-associated overactivation of pSTAT3Ser_727_ in the absence of canonical activation of pSTAT3Tyr_705_ in CLL-BC but not N-BC. It provides substantial evidence that the same stromal signals are differentially translated by CLL-BC and N-BC. Thus, stromal support of leukemia cells correlates with major intrinsic qualitative changes in CLL-BC signaling pathways leading to pSTAT3Ser_727_ overactivation, as compared with N-BC. Our data show that this pSTAT3Ser_727_ overactivation is not associated with massive STAT3 delocalization. Indeed, immunolabelings of human normal and leukemic B cells detected the presence of reasonable levels of Mt STAT3, which is confirmed by subcellular fractionation. This suggests that constitutive phosphorylation of STAT3Ser_727_ occurs within the mitochondria of CLL-BC. As opposed to our data, previous reports showed that a minor proportion of total STAT3 localizes to mitochondria upon fractionation of normal cells.^11,12,36^ One interpretation of this discrepancy is that part of Mt STAT3 is lost upon mitochondria isolation based on different subcellular fractionation protocols. Also, CLL-BC contain significantly more mitochondria as compared with N-BC. Mt STAT3 of CLL-BC might therefore be more abundant and/or less labile as compared with other cells. Also, our data indicate that nuclear pSTAT3Ser is undetectable and unable to bind STAT3-DNA-specific probes in CLL-BC. This is in contradiction with the previously reported nucleocytoplasmic trafficking of pSTAT3Ser_727_ in CLL-BC, despite the use of an identical STAT3-DNA probe.^14^ Again, different subcellular fractionation and/or pull-down protocols may explain such a discrepancy.

Overall, our findings indicate that Mt pSTAT3Ser_727_ overactivation is linked to leukemic B cell-extended survival and antioxidant defense. It suggests that pSTAT3Ser_727_ contributes to the antioxidant defense and protection of the leukemic cells. This activity may similarly reduce that of ROS-generating anticancer agents and favor drug resistance. These results suggest that pSTAT3Ser_727_ detection could be used as a new tool to characterize patients' leukemic cells from blood samples, which, together with current disease criteria, could help define new patient subgroups and help predict disease outcome and/or drug resistance. It further supports the idea of therapeutically targeting pSTAT3Ser_727_ activity to deregulate leukemic cell antioxidant defense and eliminate the CLL-BC protected by their microenvironment, which could be resistant to immune chemotherapy. To our knowledge, CLL is the first human malignancy linked to a non-canonical mitochondria-restricted pSTAT3Ser_727_ overactivity *in vivo*, thereby offering a new potential therapeutic target that spares healthy B cells.

## Materials and Methods

### Primary cells and cell lines

After obtaining the approved informed consent of Institutional Review Board, peripheral blood mononuclear cells were collected from healthy donors and CLL patients, and were isolated by density-gradient centrifugation on Ficoll Hypaque. B cells were further purified using CD3/CD14 immunomagnetic-bead-negative selection (Miltenyi Biotec, Cologne, Germany) to avoid B-cell activation. CD5/CD19 double-positive cells were selected by FACS. All patients had immunophenotypically defined CLL as outlined by the modified 1996 National Cancer Institute Criteria (Supplementary Table 1). The RAJI human B-cell line, the MS5 murine and HS5 human bone marrow stromal cell lines were purchased from DSMZ cell depository bank. They were cultured in RPMI (RAJI) and MEM alpha (MS5, HS5) medium supplemented with 10% fetal calf serum (FCS). B cells were cultured *in vitro* alone or with a layer of HS5 or MS5 cells under normoxia in RPMI medium supplemented with 5–10% FCS. The cells were incubated with GSH (2 mM) or NAC (1 mM) for 4 days and exposed to PEITC (0–5 μM) during the last 5 h of incubation. Alternatively, B cells were exposed to PEITC (0–5 μM) alone for 0–5 h. Where indicated, CLL-BC were cultured alone for 2 days in the absence or presence of the MEK inhibitor PD0325901 (0–100 nM).

### Lentiviral constructs and transduction

Oligonucleotides targeting firefly luciferase (shL, 5′-CGTACGCGGAATACTTCGA-3′), STAT3 (shS3, 5′-AAGAAACTGGAGGAGTTGCAG-3′) and STAT5A/B (shS5, 5′-GGAGAACCTCGTGTTCCTG-3′) were inserted downstream of the H1 promoter into an HIV-SIN-SFFVGFP vector that encodes GFP as reporter. Lentiviral particles displaying measles virus glycoproteins were produced and titrated on 293T cells using GFP FCM as described. B cells were incubated with lentiviral particles at a multiplicity of infection of 3 in RPMI1640 supplemented with 10% FCS for 18–24 h and then transferred on MS5 stromal cells for at least 8 days. Transduction efficiencies ranged between 10 and 30%.

### Flow cytometry measurements

CLL-BC were identified by anti-CD5-APC-Cy7 and anti-CD19-PC7 antibodies. Apoptosis was assessed by Annexin V labeling. MTP measurements were achieved using 3.3′dihexyloxacarbocyanine iodide (Di0C6(3)) or TMRM fluorescent probes, sequestered by active mitochondria.^37^ Dead cells were stained by 7-AAD or TO-PRO-3 iodide (Life Technologies, Carlsbad, CA, USA). ROS were evaluated using the DHE fluorescent probe that binds to superoxide anions. For STAT3 and pMEK labeling, CD5^+^CD19^+^ pre-labeled B cells were fixed, permeabilized and incubated with relevant antibodies, according to Irish *et al.*^38^ The antibodies were total STAT3, pSTAT3Tyr_705_, pSTAT3Ser_727_ and pMEK (BD Biosciences, San Jose, CA, USA). pSTAT3-Ser_727_ antibodies were pre-mixed or not with pSTAT3-Ser_727_ ipep (Santa Cruz, Dallas, TX, USA) or a chemically synthesized STAT3 peptide (IDLPMS_727_PRTLD) that was phosphorylated (pS3-11pep) or not (S3-11pep) on Ser_727_ (Thermo Fisher Scientific, Waltham, MA, USA) at a 1 : 100 molar ratio. FCM analyses were performed using Diva and Cellquest on a FACS-Canto II (BD Biosciences). Data are expressed as the percentage of labeled cells among total or as the mean fluorescence intensity.

### Immunofluorescence microscopy

B cells, pre-incubated or not for 15 min at 37 °C with red Mitotracker (500 nM, Life Technologies) were loaded on poly-L-lysine-coated slides, fixed in 3.7% paraformaldehyde, permeabilized with phosphate-buffered saline (PBS)/0.1% Triton X-100 for 5 min at room temperature and incubated in 1 × PBS/5% bovine serum albumin (BSA) for 1 h at 37 °C in the presence of primary antibodies. The latter were murine anti-pSTAT3Ser_727_ antibodies (BD Biosciences), rabbit anti-pSTAT3Ser_727_ and anti-total STAT3 antibodies (Cell Signaling Technology, Danvers, MA, USA), and antibodies directed to the Mt DNA-encoded complex I (NADH-ubiquinone reductase) subunit (mtND1, gift of Dr. Lombes).^16^ Rabbit pSTAT3-Ser_727_ antibodies were pre-mixed or not with the indicated pSTAT3-Ser_727_ peptide (Santa Cruz) at a 1 : 1 concentration ratio. Then, FITC-conjugated anti rabbit immunoglobulin G antibody (BD Biosciences) or PE-conjugated anti-murine immunoglobulin G (Life Technologies) secondary antibodies were used for 1 h incubation at room temperature. Slides were DAPI-stained and viewed using a Leica confocal microscope (LEICA,Wetzlar, Germany), with a digital AxioCamERc camera and analyzed with AxioVisio software (Carl Zeiss, Jena, Germany). At least 25 cells/patient sample were observed at × 63 and × 100 magnifications. Co-localization analyses used a cooled Coolsnap camera (Roper Scientific, Trenton, NJ, USA) with optical *z*-sections collected at 0.63 *μ*m steps. Image analyses were performed with the software ImageJ associated with the JACoP plugin tool to integrate statistics to spatial exploration of correlated pixel signals (expressed as Pearson's correlation coefficient). Coefficients >0.5 indicated significant colocalization.^39^

### Transmission electron microscopy

B cells were fixed in 3% glutaraldehyde and postfixed in 1 × PBS/1% osmium tetroxide, dehydrated in ethanol followed by propylene oxide (VWR, Radnor, PA, USA), embedded in Epon and polymerized overnight at 60 °C. Ultrathin sections were cut with a LEICA/Reichert Ultracut S ultramicrotome, stained with uranyl acetate and lead citrate. Immunolabeling was performed on B cells fixed in 0.2% glutaraldehyde/4% paraformaldehyde for 1 h at room temperature as described,^40^ using rabbit total STAT3 or pSTAT3Ser_727_ antibodies (Cell Signaling Technology). Ultrathin (Peabody, MA, USA) sections were examined with a JEOL1011 transmission electron microscope. Digital images of ≥10 cells/sample were obtained using a GATAN-CCD camera and Digital Micrograph software (Warrendale, MA, USA).

### Preparation of mitochondria extracts

Mitochondria were isolated from B cells using Mitochondria isolation kit (Miltenyi Biotec) following manufacturer's instructions with some modifications. Briefly, frozen B cells (2–10 × 10^7^ cells) were directly resuspended in pre-cooled phosphate-buffered saline (1 ml per 10^8^ total cells) supplemented with ethylenediamine tetraacetatic acid (EDTA; 2 mM), anti-protease and –phosphatase inhibitors and benzonase (50 U), and incubated for 20 min at 4 °C. Cell homogenization was performed with a 26-G needle stepwise using 5–10 stokes. Lysates were diluted to 1 × separation buffer (Miltenyi Biotec) and proceeded to magnetic labeling by incubation with anti-Tom22 magnetic beads for 1 h at 4 °C on a wheel. The labeled cell lysate was loaded on a MACS column placed in a magnetic field and let run through. Lysates were re-loaded three times. Column was then intensively washed out and the magnetically labeled mitochondria were then flushed out. Pre- and post-mitochondria-purified fractions were separated by SDS-PAGE and the presence of cytoplasmic *β-*actin protein, Mt hsp60 or NDUFA9 proteins, and nuclear KDM1a were evaluated by western blot analysis using monoclonal anti-*β*-actin (AC-15, Sigma-Aldrich), -hsp60 (Biosciences Inc., Allentown, PA, USA), -NDUFA9 (Abcam, Cambridge, UK) and KDM1a (Cell Signaling Technology).

### Immunoprecipitation

Total cell extracts from B cells (5 10^7^) were obtained by a 30-min incubation at 4 °C in lysis buffer (20 mM Tris, pH 8, 137 mM NaCl, 1% NP40, 10% glycerol and 2 mM EDTA) supplemented with cocktails of proteinase (Roche) and phosphatase inhibitors. Lysates were immunoprecipitated for 4 h at 4 °C with 4 *μ*g of complex I immunocapture monoclonal antibody (MitoSciences, Eurogen, OR, USA) or isotype-matched control (ctl). Immunoprecipitates were separated by SDS-PAGE and immunoblotted with the indicated antibodies.

### Oligo pull-down assays

Cytoplasmic and nuclear extracts were obtained from B cells, as previously described.^41^ To confirm proper subcellular fractionation, extracts were separated by SDS-PAGE and the presence of the nuclear lamin B and A/C, or the cytoplasmic *β*-actin was assessed by western blot analysis using monoclonal mouse anti-*β*-actin (Sigma-Aldrich), polyclonal rabbit anti-lamin B, -lamin A/C (Cell Signaling Technology) antibodies. Nuclear extracts (2–5 × 10^7^) were incubated with 1 μg of the indicated biotin-labeled STAT3-DNA probe SIE367 (5′-TTCCCGTAA-3′) or IRF1 (5′-GATCCATTTCCCCGAAATGA-3′) or control Oct-DNA probe (5′-CGGGTATAATTTCTGC-3′) in binding buffer (10 mM HEPES, 100 *μ*M EDTA, 50 mM NaCl, 50 mM KCl, 5 mM MgCl_2_, 4 mM spermidine, 2 mM dithiothreitol, 0.1 mg of BSA/ml, 2.5% glycerol and 4% Ficoll) for 2 h at 4 °C, as described.^41^ Streptavidin magnetic beads (Pierce, Thermo Fisher Scientific) were added for 30 min at 4 °C, extensively washed, resuspended in SDS-PAGE loading buffer and analyzed by SDS-PAGE and immunoblotting.

### Statistics

Data were expressed as means or medians depending on the normality of the distribution. Statistical significance was determined by Student's *t*-test or Mann–Whitney/Wilcoxon test. *P*-values≤0.05 were considered as significant (*) and *P*-values ≤0.01 as highly significant (**). Statistical analyses were performed with the Medcalc (Ostende, Belgium) and GraphPad Prism softwares (San Diego, CA, USA).

## Abbreviations

CLL: chronic lymphoid leukemia
CLL-BC: CLL B lymphoid cells
N-BC: normal B lymphoid cells from healthy donors
S3: STAT3
pSTAT3Ser727: Ser727-phosphorylated STAT3
pSTAT3Tyr705: Tyr705-phosphorylated STAT3
MTP: mitochondria transmembrane potential
FCM: flow cytometry measurement
7-AAD: 7-aminoactinomycin D
TMRM: tetramethylrhodamine-methyl-ester
Mt: mitochondrial
ND1: NADH-ubiquinone reductase subunit
DHE: dihydroethidium
PEITC: *β*-phenylethyl isothiocyanate
NAC: N-acetyl cysteine
GSH: glutathione
