# Supplementary material for: Viability and stress protection of chronic lymphoid leukemia cells involves overactivation of mitochondrial phosphoSTAT3Ser727
Source: Cell Death Dis. 2014 Oct 9;5(10):e1451–. doi: 10.1038/cddis.2014.393 (PMC4237234; doi:10.1038/cddis.2014.393)
Supplement: Supplementary Information [file cddis2014393x1.pdf]

Capron et al, Supplementary Data.

This supplementary file contains:

- Supplementary Table S1. CLL patient characteristics
- Supplementary Figure S1-S3 files. S1, circulating CLL B-cells constitutively express phosphorylated $STAT3Ser_{727}$ in absence of STAT3 and STAT5 tyrosine-phosphorylation; S2, pSTAT3Ser727 immunolabeling is suppressed in the presence of phosphorylated but not by unphosphorylated S3-11 peptide; S3, HS5 and MS5 stromal cells sustain CLL-BC viability and $pSTAT3Ser_{727}$ over-activation.

Table 1. Patients characteristics

**Patient ID**	**Time after diagnosis (ye)**	**Age (ye)**	**Hb (g/dL)**	**Platelets $10^9/L$**	**Lymp1 $10^9/L$**	**Binet stage**	**Matutes score**	**Lymph double time**	**Lymph node area**	**HSM**	**cytogenetic**	**CD19/ CD38 %**	**ZAP %**
**1**	4	72	14.1	199	10.35	A	5	>1	0	-	-	1	1
**2**	4	74	14.8	166	35.63	A	5	>1	2	Yes	normal	4	7
**3**	8	77	13.4	128	22.15	A	?	>1	0	No	-	2	2
**4**	26	79	14.6	161	36.31	A	5	>1	1	-	normal	NEG	1
**5**	8	68	14	156	7.58	A	5	>1	0	no	del chrom. 2 ; chrom 11 rearrangement	1	5
**6**	4	66	17.1	211	9.11	A	4	>1	0	-	-	2	7
**7**	0	76	13.1	176	8.10	A	4	-	0	-	-	1	NEG
**8**	?	93	9	118	155.00	C	?	>1	>3	Yes	-	86	22
**9**	11	73	14.4	173	21.50	A	5	>1	0	No	-	10	NEG
**10**	2	90	14.9	158	21.84	A	?	>1	2	no	-	71	NEG
**11**	8	77	9.5	46	110.27	C	5	<1	>3	Yes	-	37	9
**12**	3	67	14.1	174	19.86	A	4	>1	0	-	-	1	1
**13**	2	60	12.3	178	10.30	A	5	<1	0	No	-	2	3
**14**	?	66	14	146	7.32	A	5	<1	1	No	-	0,5	4
**15**	1	65	16.7	128	11.63	A	4	>1	-	-	Y loss	2	1
**16**	8	50	15.5	139	17.40	A	5	>1	-	-	normal	0,5	3
**17**	6	83	12.4	309	6.31	A	5	>1	0	-	-	3	0.5
**18**	5	69	13.1	225	9.00	A	3	>1	-	-	Trisomy 12, 18; monosomy X	62	3
**19**	6	53	13.6	176	51.32	A	?	>1	0	Yes	-	0	8
**20**	8	59	13.8	129	3.23	A	5	>1	0	No	normal	1	10
**21**	2	83	12 .6	258	34.70	A	5	>1	0	?	-	3	4
**22**	0	74	14.1	287	5.00	A	5	-	0	No	normal	3	NEG
**23**	2	79	12.9	208	68.70	B	5	<1	>3	Yes	Del 11q	NEG	1
**24**	0	63	14.6	217	11.31	A	4	-	1	No	Del 13q ; t(13)	3	2
**25**	0	80	15.4	181	3.74	A	4	>1	0	No	-	7	3
**26**	1	70	16.2	221	11.00	A	5	>1	0	No	-	1	1
**27**	0	75	14.7	240	11.00	A	5	-	0	No	-	16	NEG
**28**	-												
**29**	1	59	15.3	174	11.79	A	4	>1	0	No	normal	1.5	3
**30**	?	71	14	300	6.50	A	5	>1	0	No	-	0	7

Supplementary Table I. CLL patient characteristics. All patients had not received any treatment for at least 2 years prior the study. Hb, hemoglobin; HSM, hepatospenomegaly; - indicates not determined.

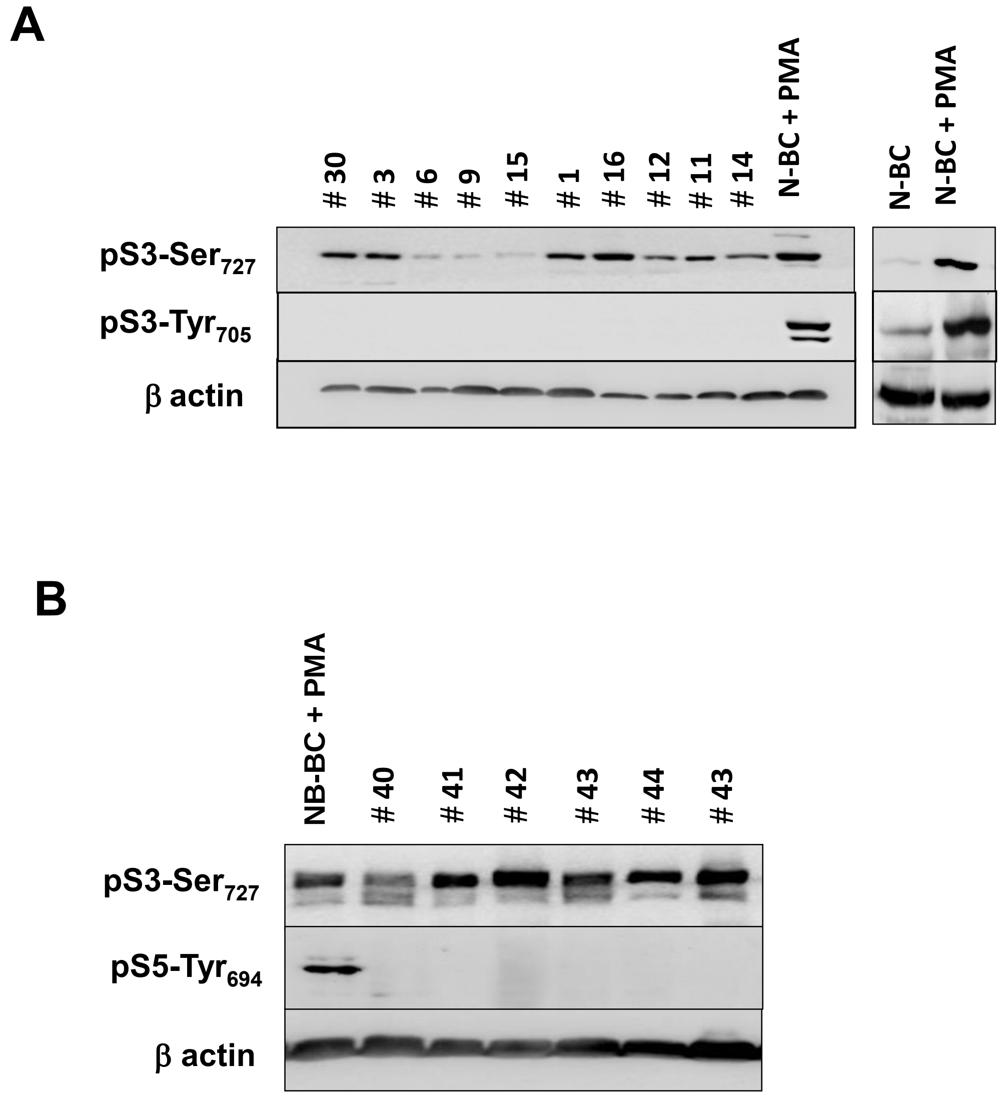

Supplementary Figure S1. Circulating CLL B-cells over-express phosphorylated STAT3Ser$_{727}$ in absence of STAT3 and STAT5 tyrosine-phosphorylation. Western blot analysis of freshly isolated CLL B-cells (CLL-BC, n=16) and normal B-cells (N-BC, n=3) using the indicated antibodies. Where indicated, N-BC were stimulated with phorbol myristyl acetate (PMA, 10 µM) for 15 min as a positive control. #n identifies individual patient.

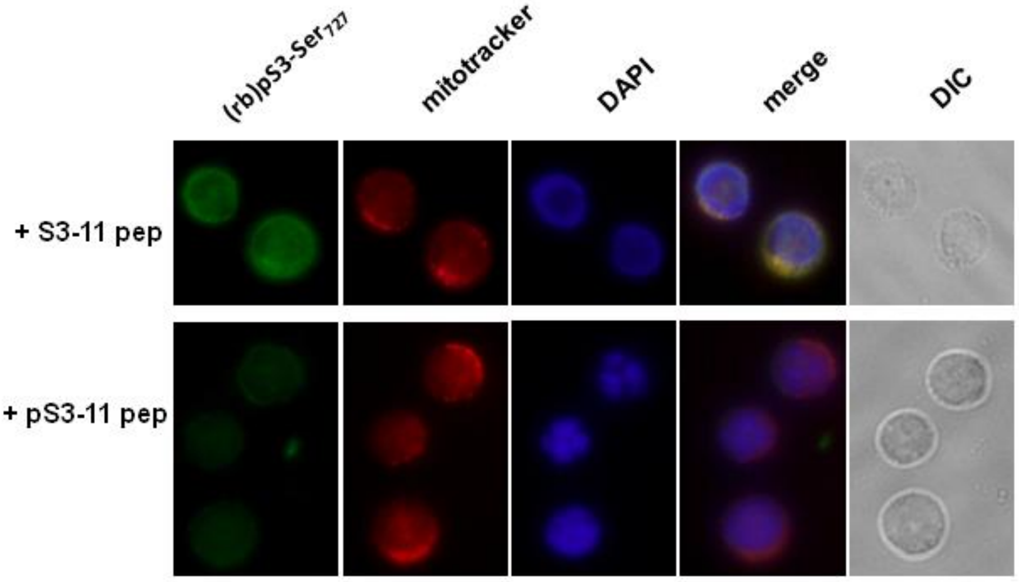

Supplementary Figure S2. pSTAT3Ser$_{727}$ immunolabeling is suppressed in the presence of phosphorylated but not unphosphorylated S3-11 peptide. CLL-BC were isolated, fixed, permeabilized and processed for fluorescence microscopy using the indicated primary antibody and fluorescent probes. B cells were labeled with rabbit pSTAT3Ser$_{727}$ antibody in the presence of unphosphorylated (S3-11pep; upper panel) or phosphorylated (p-S3-11pep; lower panel), as indicated. Fluorescent probes were FITC-coupled anti-rabbit Ig (green); mitotracker (red) and DAPI (blue). DIC stands for differential interference contrast. (magnification x100).

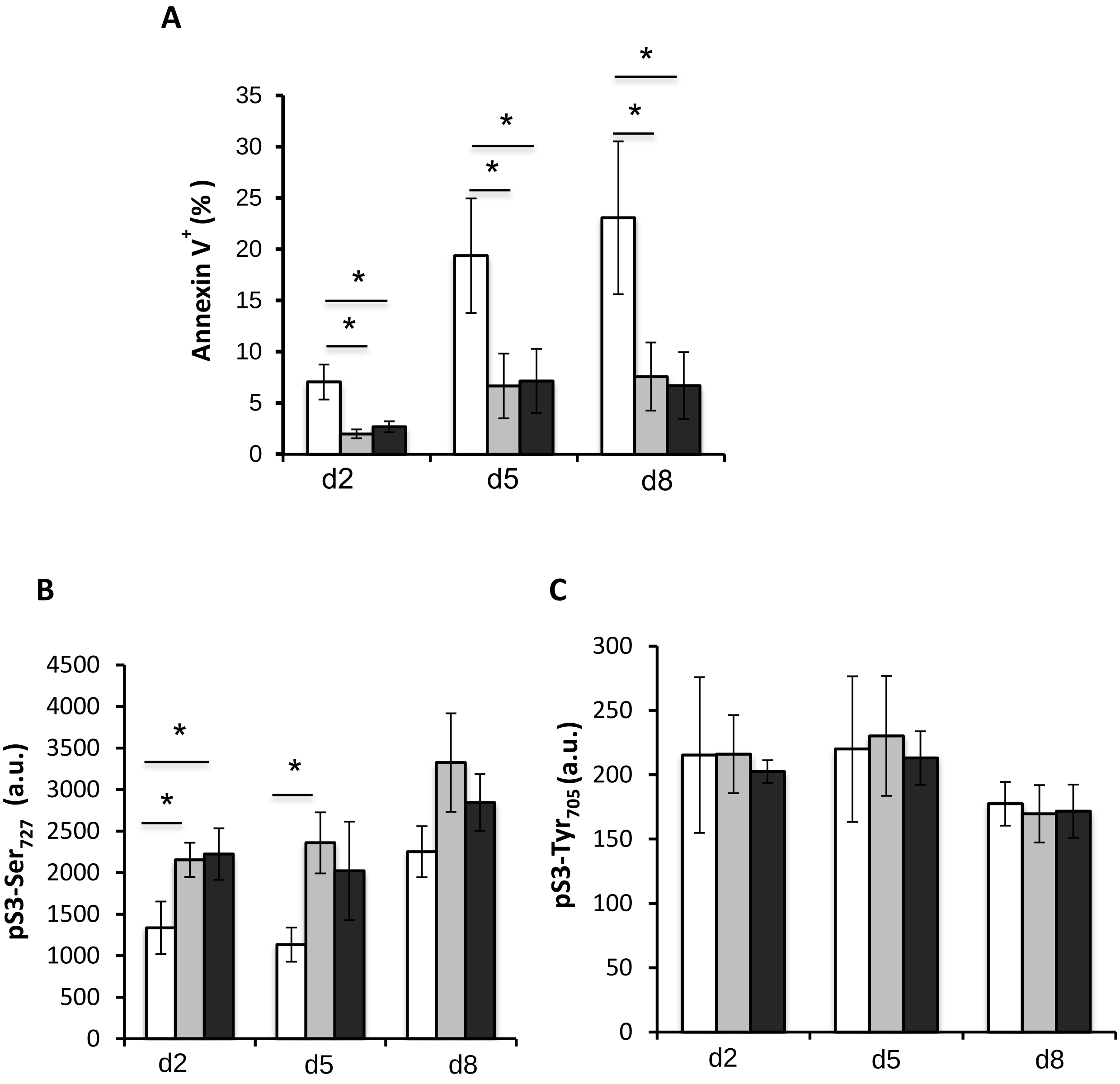

Supplementary Figure S3. Human and murine stromal cells sustain CLL cell viability and pSTAT3Ser$_{727}$ over-activation. FCM analysis of Annexin V (A), pSTAT3Ser$_{727}$ (B) or pSTAT3Tyr$_{705}$ (C) expression of CLL-BC along time of culture. CD45^{+}CD19^{+} CD5^{+}B-cells were analyzed at the indicated time (days, d) upon liquid culture (white bars), or co-culture in the presence of either MS5 (grey bars) or HS5 (black bars). Standard rabbit immunoglobulins were used as control. Results are expressed as percentage of AnnexinV positive cells (A) or mean fluorescence intensity (arbitrary unit, a.u.) (B-C) (mean ± SEM, n=3; * $p<0.05$).
